# Retraction: Neuroprotective Role of Intermittent Hypobaric Hypoxia in Unpredictable Chronic Mild Stress Induced Depression in Rats

**DOI:** 10.1371/journal.pone.0319289

**Published:** 2025-02-07

**Authors:** The PLOS One Editors

After publication of this article [1], concerns were raised about results presented in Figs 9, 10, and 12. Specifically, the following panels appear to fully or partially overlap despite representing different experimental conditions:

- Fig 9 CONTROL+IMIP and Fig 12 DCX UCMS+IHH+Veh
- Fig 10B CONTROL, CONTROL+IHH, and UCMS+IMIP
- Fig 10B CONTROL+IMIP, Fig 10B UCMS+IHH, and Fig 12 BDNF UCMS+IHH+Veh at different magnification

The corresponding author stated that the Fig 12 DCX UCMS+IHH+Veh panel is incorrect and that the underlying image data for the panels in Fig 10B are no longer available. They provided partial underlying data for Figs 9, 10, and 12, and replacement panels for Fig 10B and for the DCX UCMS+IHH+Veh and BDNF UCMS+IHH+Veh panels in Fig 12, but the *PLOS One* Editors considered that these did not resolve the original concerns.

In light of the above unresolved concerns that question the integrity and reliability of the reported results and conclusions, the *PLOS One* Editors retract this article.

NKushwah, VJ, SD, DP, and SBS either did not respond directly or could not be reached. NKhan responded but expressed neither agreement nor disagreement with the editorial decision.
